# Molecular Epidemiology in Amerindians of the Brazilian Amazon Reveals New Genetic Variants in DNA Repair Genes

**DOI:** 10.3390/genes13101869

**Published:** 2022-10-15

**Authors:** Amanda de Nazaré Cohen-Paes, Angélica Leite de Alcântara, Fabiano Cordeiro Moreira, Marianne Rodrigues Fernandes, Karla Beatriz Cardias Cereja Pantoja, Darlen Cardoso de Carvalho, João Farias Guerreiro, Ândrea Ribeiro-dos-Santos, Sidney Emanuel Batista dos Santos, Paulo Pimentel de Assumpção, Ney Pereira Carneiro dos Santos

**Affiliations:** 1 Oncology Research Nucleus, Universidade Federal do Pará, Belém 66073-000, Pará, Brazil; 2 Human and Medical Genetics Laboratory, Instituto de Ciências Biológicas, Universidade Federal do Pará, Belém 66075-110, Pará, Brazil

**Keywords:** DNA repair, Native American, Brazil, genetic variants, continental populations

## Abstract

Native American populations from the Brazilian Amazon have a low genetic diversity and a different genetic profile when compared to people from other continents. Despite this, few studies have been conducted in this group, and there is no description of their genetic data in the various currently existent international databases. The characterization of the genomic profile of a population not only has an impact in studies of population genetics, but also helps to advance diagnostic and therapeutic response studies, leading to the optimization of clinical applicability. Genetic variations in DNA repair genes have been associated with the modulation of susceptibility to various pathologies, as well as in their prognosis and therapy. This is the first study to investigate DNA repair genes in Amerindians from the Brazilian Amazon region. We investigated 13 important DNA repair genes in the exome of 63 Native Americans, comparing our results with those found in 5 continental populations, whose data are available in the Genome Aggregation Database. Our results showed that 57 variants already described in literature were differentially distributed in the Amerindian populations in relation to the continental populations, 7 of which have significant clinical relevance. In addition, 9 new variants were described, suggesting that they are unique to these populations. Our study reinforces the understanding that the Amazonian Native American population presents a unique genetic profile, and our findings may collaborate with the creation of public policies that optimize the quality of life of these groups as well as the Brazilian population, which presents a high degree of interethnic mixing with Amerindian groups.

## 1. Introduction

Genome integrity depends not only on faithful replication, but essentially on proper repair of the data in DNA throughout its synthesis and processing. It is estimated that approximately 10,000 DNA bases are chemically modified every day in each cell [1,2]. These modifications can be spontaneous or induced by chemicals or radiation, causing genomic mutations such as nucleotide substitutions, amplifications, deletions, rearrangements, or chromosomal loss [3,4]. In order to avoid these mutations, cells have evolved different DNA repair mechanisms to respond to specific damage and prevent mutagenesis [5,6,7]. There are at least four DNA repair mechanisms that have been well-described in humans: base excision repair (BER), nucleotide excision repair (NER), homologous recombination repair (HRR), and the mismatch/mismatch repair (MMR) pathway [8,9,10,11].

Given the importance of their functions, several studies have associated genes of DNA repair pathways and susceptibility to diseases such as xeroderma pigmentosum [12,13], cockayne syndrome and trichothiodystrophy [13], and cancer [14,15,16,17]. Additionally, when it comes to cancer, polymorphisms in these genes have been shown to be linked not only to its development, but also to the mechanism of resistance to pharmacological treatment [16,18,19,20,21].

Despite the amount of information available in literature regarding imbalances in gene expression of DNA repair genes, there are no studies reporting the frequency of mutations in these genes in Amerindian populations, especially in the Amazon. However, studies of precision medicine have demonstrated the importance of screening not only genetically homogeneous populations, such as the European one, but also Amerindian people distributed worldwide. It has been shown that populations with a high prevalence of Amerindian genomic ancestry have an increased predisposition to develop acute lymphoblastic leukemia, stomach cancer, and tuberculosis [22,23,24,25].

Thus, genomic studies on DNA repair genes in Amerindian populations may provide information on their molecular profile and may potentially discover new variants. The findings from molecular epidemiology studies may also promote the investigation of clinical implications regarding the molecular markers described, allowing them to be used as diagnostic and treatment tools for the Amerindian population, as well as for the admixed populations with marked Amerindian ancestry, such as the Brazilian one [26]. Finally, findings from this type of data are the basis for inferences about human evolutionary history [27]. The aim of this study is to characterize the molecular profile of genes present in DNA damage repair pathways in Amerindian populations from the Brazilian Amazon, and to compare these findings with data from continental populations described in the Genome Aggregation Database (gnomAD).

## 2. Materials and Methods

### 2.1. Study Population and Ethics

The study was approved by the National Research Ethics Committee (CONEP; available at: http://conselho.saude.gov.br/comissoes-cns/conep/) and by the Research Ethics Committee of the Tropical Medicine Center of the Federal University of Pará (CAE: 20654313.6.0000.5172). All individuals and community leaders signed an informed consent form.

The study population consisted of 63 Amerindians from the Brazilian Amazon. Amerindians represent 12 different Amazonian ethnic groups: Asurini do Xingu, Arara/Arara do Iriri, Araweté, Asurini do Tocantins, Awa-Guajá, Kayapó/Xikrin, Zo’é, Wajãpi, Karipuna, Phurere, Munduruku, and Yudjá/Juruna. These individuals were grouped into a single sample named NAT (Native American population).

The Amerindian population data were compared with representatives of five continental populations obtained from the Genome Aggregation Database (available at: https://gnomad.broadinstitute.org/; accessed on 15 February 2022), a public catalog of human variation and genotype data. This sample included 8128 individuals from Africa (AFR), 56,885 from Europe (EUR non-finish), 17,296 from the Americas (AMR), 9197 from East Asia (EAS), and 15,308 from South Asia (SAS).

### 2.2. Extraction of the DNA and Preparation of the Exome Library

DNA was extracted from a peripheral blood sample using the phenol-chloroform method described by Sambrook et al. [28]. To quantify the genetic material, the Nanodrop-8000 spectrophotometer (Thermo Fisher Scientific Inc., Wilmington, DE, USA) was used and its integrity was evaluated by 2% agarose gel electrophoresis.

Libraries were prepared using the Nextera Rapid Capture Exome (Illumina^®^, San Diego, CA, USA) and SureSelect Human All Exon V6 (Agilent technologies, Santa Clara, CA, USA) kits, following the manufacturer’s recommendations. The sequencing reactions were performed on the NextSeq 500^®^ platform (Illumina^®^, San Diego, CA, USA) using the NextSeq 500 High-Output v2 Kit 300 cycle kit (Illumina^®^, San Diego, CA, USA).

### 2.3. Bioinformatic Analysis and Statistical Analyses

Reads in FASTQ format were analyzed for quality (FastQC v.0.11 http://www.bioinformatics.babraham.ac.uk/projects/fastqc/, accessed on 25 June 2021) and filtered to eliminate low-quality reads (fastx_tools v.0.13 http://hannonlab.cshl.edu/fastx_toolkit/; accessed on 25 June 2021). Then, the sequences were aligned with the reference genome (GRCh38) using the BWA v.0.7 tool (http://bio-bwa.sourceforge.net/; accessed on 20 March 2022).

After alignment, the generated file was indexed and sorted (SAMtools v.1.2—http://sourceforge.net/projects/samtools/; accessed on 20 March 2022). The alignment had to be processed to remove duplicate readings (Picard Tools v.1.129—http://broadinstitute.github.io/picard/; accessed on 20 March 2022), mapping quality recalibration and local realignment (GATK v.3.2—https://www.broadinstitute.org/gatk/). Finally, the result was processed in search of variants (GATK v.3.2, United States; https://gatk.broadinstitute.org/hc/en-us) of the reference genome. The allelic variants were annotated in the ViVa^®^ (Viewer of Variants, Natal, RN, Brazil) software. Markers in PCLO gene were also selected in coverage, where the readout should be high-coverage, with a minimum of 10 reads (fastx_tools v.0.13—http://hannonlab.cshl.edu/fastx_toolkit/; accessed on 20 March 2022).

All statistical analyses were performed using the R Studio v.3.5.1 program (R Foundation for Statistical Computing, Vienna, Austria), including the discriminant analysis of principal components (DAPC). Significant differences in allele frequencies between populations were analyzed by Fisher’s exact test. The false discovery rate (FDR) proposed by Benjamini and Hochberg [29] was used to correct the multiple analyses. Results were considered statistically significant when the p-value was less or equal than 0.05 (*p* ≤ 0.05).

### 2.4. Selection of the Genetic Variations

We analyzed 13 gene components of 4 different DNA repair pathways in humans (base excision repair (BER), nucleotide excision repair (NER), homologous recombination repair and mismatch repair). A total of 432 variants were found, to which the following selection criteria were applied: (I) the read should be high-coverage, with a minimum of 10 reads (fastx_tools v.0.13—http://hannonlab.cshl.edu/fastx_toolkit/; accessed on 25 March 2022); (II) the predicted impact should be “modifier”, “moderate”, or “high” according to the software SNPeff (https://pcingola.github.io/SnpEff/; accessed on 25 March 2022); (c) the difference in allelic frequency of the variants between NAT populations and continental populations should be significant (*p*-value ≤ 0.05).

## 3. Results

After applying our selection criteria to the data from the complete exome of the 63 Amerindians investigated, 55 genetic variants were analyzed, which were distributed in 13 genes composing the DNA repair pathways in humans: DNA2, NEIL1, NEIL2, NEIL3, TOP3A, XPC, XRCC1, XRCC3, ERCC1, ERCC2/XPD, ERCC5, MSH3, and MSH4. Table 1 presents the genetic variants and their respective specifics: chromosomal location, genomic position, the wild allele and the mutant allele, the detailed genomic region, gene, and SNPId. We also find the allele frequency data for the world populations investigated here, and the respective allele frequency calculated for variants in the Amerindian population (NAT).

Thirteen of the investigated variants were differentially distributed in the NAT population when compared to the five continental populations investigated, among them: rs10823209 (DNA2); rs7689099 (NEIL3); rs2294913 (TOP3A); rs3212038, rs1799796, rs861531, and rs861537 (XRCC3); rs6151734 and rs1105524 (MSH3); rs3765682 (MSH4); rs907187, rs2293464, and rs1805404 (PARP1). Overall, for the other SNVs presented, the Amazonian indigenous population differed from two or three of the investigated world populations.

**Figure 1:**
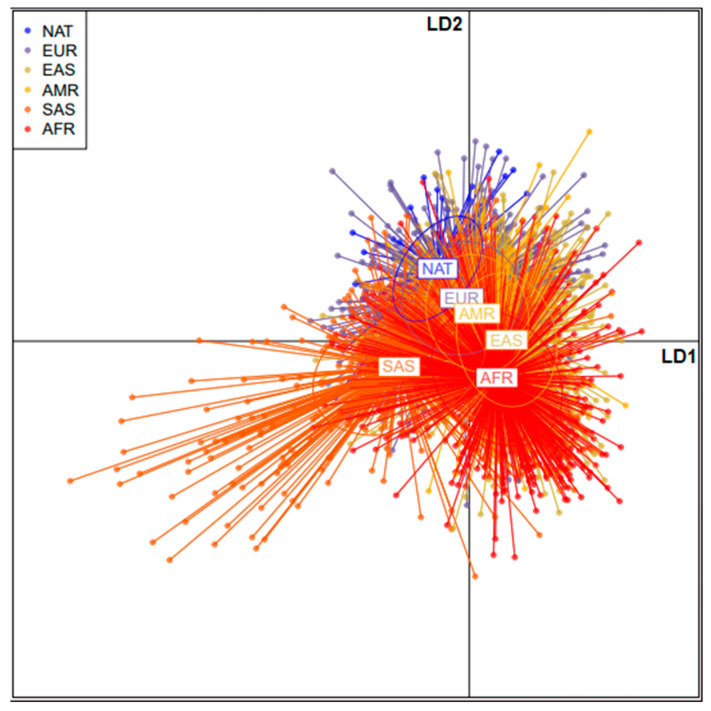
Discriminant analysis of principal components (DAPC) of the analyzed variants in the selected repair genes for Native American populations (NAT), African population (AFR), European population (EUR), American population (AMR), East Asian population (EAS), and South Asian population (SAS).

Figure 1 shows a discriminant analysis of principal components (DAPC) scatterplot of the six populations analyzed. The DAPC analysis allows for the visualization of well-defined clusters according to the similarity or difference of the frequency distributions of each investigated SNV. Figure 1 demonstrates a significant distance between the Amerindian population (NAT) and the African population.

Table 2 shows the pairwise analysis between the allele frequencies in Amerindians and each of the five continental populations of the gnomAd database for each variant of the repair genes investigated, seven of which have a clinical impact for the development of some pathology or on the therapeutic efficacy of some pathway in question according to the ClinVar database (National Center for Biotechnology Information—NCBI), and three of which have a high impact according to the SNPeff software. For rs1799796 (XRCC3) and rs184967 (MSH3), the NAT population showed a significantly different distribution to the frequencies in relation to American and European populations, these SNVs being respectively associated with susceptibility to breast cancer and hereditary cancer predisposition syndrome.

Supplementary Table S1 shows the same analysis as Table 2 on the 48 markers that are statistically significant, but not clinically significant according to the classification available in the ClinVar database (NCBI), nor classified as high-impact.

Finally, Table 3 describes the chromosomal/genomic position data, the wild and mutant alleles, the impact, type of genetic variation, protein exchange that the mutation may entail, detailed region, and repair pathway that these markers comprise in nine novel genetic variants found in the investigated Amerindian populations. These variants have never been described in the literature and are present in DNA2, PARP2, TOP3A, ERCC2, ERCC5, and MSH3 genes, of which one has a predicted high impact, one has a modifier impact, and the others have a moderate impact.

For all missense variants, we used two other impact prediction tools (SIFT and PolyPhen). Table 4 describes this data for each possible gene transcript in the investigated genomic region.

## 4. Discussion

The study of genetic variations in repair genes has aided the understanding of various pathological mechanisms, describing risks associated with both individual and population genotypes based on the exposure of cells to xenobiotics that lead to genomic instability [10,30,31,32,33,34,35,36,37].

The past process of global colonization is known to have played an important role in defining current patterns of genetic diversity, and it partially explains geographic variation in susceptibility to certain complex diseases [38,39]. Thus, predisposition to certain illnesses may have an intrinsic relationship with genomic ancestry [40,41,42]. Recent investigations have shown that Brazilian Amerindian populations have a unique genetic profile, yet it remains undescribed in major population databases [23,43,44].

Therefore, we analyzed the complete exome of 13 DNA repair genes never previously investigated in Amerindian populations, representative of tribes from the Brazilian Amazon. Our description of frequencies and our DAPC analysis demonstrated that the African, South Asiatic, and Amazonian Native American populations were positioned at opposite extremes, being the most genetically distinct regarding the investigated variants, corroborating with findings on the history of human populations [45]. Our results also showed that at least seven of the investigated variants had a high clinical impact, both in disease predisposition and in modulating therapeutic response. The markers rs1799796 of the XRCC3 gene and rs184967 of the MSH3 gene were shown to be statistically significant regarding their distribution in the NAT population when compared to the AMR and EUR ones. The XRCC3 gene encodes a protein involved in the HRR pathway and in the repair of strand breaks caused by X-rays [11,46]. Based on the function of XRCC3, mutations in this gene are related to the development of various neoplastic types, such as osteosarcoma [47], bladder cancer [48], and thyroid cancer [9], among others [11]. The rs1799796 investigated here is primarily associated with susceptibility to breast cancer, an association reinforced by the investigation of Niu and colleagues (2021), who performed a meta-analysis of 13 major studies previously published in literature [49,50,51].

The MSH3 gene is part the MMR system, whose function is to repair DNA after cross-linking chains and recognize and correct base–base mismatches and insertion/deletion loops generated during DNA replication and homologous recombination [11,52]. The combination of genetic variants in this gene can disrupt cellular responses to DNA damage, directly influencing an individual’s sensitivity to carcinogens, so that mutations in the MSH3 gene can modulate everything from carcinogenesis and metastatic progression to therapeutic response [17,53]. The rs184967 (c.2846A>G; Gln940Arg) was associated with increased risk of proximal colon cancer (*p* = 0.005), as well as a worse progression-free survival [54]. This polymorphism is also associated with familial breast cancer [55,56] and colon and rectal cancer [54].

Finally, we found nine new variants, among which three are INDEL-like, and one—which causes a reading matrix change—has an estimated high clinical impact. This mutation is located in the TOP3A gene, responsible for homologous recombination-mediated repair of double-stranded DNA during DNA synthesis, an essential component in the mitochondrial DNA replication process [57]. The remaining novel variants found are distributed among four important DNA repair pathways in humans (Table 3). This data reinforces the importance of studying Amerindian populations, to discover the clinical impact of these mutations, since they are found in critical genes for maintaining genomic integrity. The advantages of studying populations with low genetic diversity such as ours for screening complex diseases are the high degree of linkage disequilibrium, reduced haplotype complexity, and greater potential for identifying rare variants [58,59]. Kuhn and collaborators in 2012 conducted a study analyzing the genomic profile of the Xavante tribe with other populations, including Brazilian populations, and demonstrated that the indigenous population investigated remained genetically isolated, potentially providing a unique opportunity for hereditary disease-mapping studies [22].

This is the first study to investigate DNA repair genes in Amerindian populations from the Brazilian Amazon, which are genetically unique and not yet described in any of the available databases on human genetic variability. Knowledge of different patterns in human genetic diversity is important in many areas of medical genetics, and it can be used as a tool to maximize understanding regarding susceptibility, diagnosis, prognosis, and therapeutic management for Native American populations, as well as for populations with high Amerindian ancestry, such as the Brazilian one.

## 5. Conclusions

Our study reinforces the understanding that the Amazonian Native American population presents a unique genetic profile. The characterization of repair genes in this populations is an important tool for future studies regarding their association with complex diseases in these populations and also in ones with a high degree of admixture with these groups. Furthermore, our data may contribute to the creation of public policies that optimize the quality of life of Amerindian populations investigated here.

## Figures and Tables

**Table 1:** Allele frequencies of the variants investigated in the Amerindian individuals (NAT) and continental populations (African (AFR), American population (AMR), East Asian (EAS), European (EUR), and South Asian (SAS)) described in the gnomAD database.

Chromosome	Position	Wild	Variant	Region Detailed	Gene	SNPID	AFR	AMR	EAS	EUR	SAS	NAT
chr10	68470579	A	C	Intron	*DNA2*	rs10823209	0.1702	0.1304	0.2576	0.1397	0.1443	0.0527
chr10	68446276	TA	T	Intron	*DNA2*	rs34922453	0.5121	0.1948	0.0608	0.1473	0.0572	0.0139
chr10	68414950	T	A	3′-UTR	*DNA2*	rs1801041	0.2288	0.3172	0.2342	0.3119	0.000	0.0455
chr15	75351373	T	C	Intron	*NEIL1*	rs11634109	0.09616	0.1283	0.02490	0.2639	0.06537	0.0834
chr4	177335759	C	G	Nonsynonymous coding	*NEIL3*	rs7689099	0.05816	0.1333	0.02583	0.1165	0.0900	0.000
chr4	177353596	C	T	Nonsynonymous coding	*NEIL3*	rs13112358	0.6919	0.7025	0.3976	0.7599	0.5836	0.3645
chr4	177353681	A	C	Nonsynonymous coding	*NEIL3*	rs13112390	0.7222	0.7650	0.4594	0.7902	0.6484	0.5509
chr17	18280697	G	T	Intron	*TOP3A*	rs6502644	0.3374	0.2471	0.07407	0.3158	0.1864	0.0429
chr17	18305230	G	A	Intron	*TOP3A*	rs7207123	0.6050	0.2903	0.07764	0.3230	0.1978	0.0572
chr17	18285570	C	T	Intron	*TOP3A*	rs2294914	0.09835	0.1614	0.1618	0.1037	0.1845	0.2715
chr17	18299682	C	T	Intron	*TOP3A*	rs2294913	0.1523	0.2384	0.3018	0.2434	0.1797	0.0334
chr17	18290934	C	T	Nonsynonymous coding	*TOP3A*	rs28671051	0.1744	0.03313	0.003654	0.004159	0.01035	0.000
chr17	18290697	C	A	Intron	*TOP3A*	rs3817992	0.1525	0.2388	0.3031	0.2435	0.1799	0.381
chr3	14178523	G	C	Nonsynonymous coding	*XPC*	rs1870134	0.005	0.09175	0.2159	0.005	0.03741	0.2937
chr14	103711849	A	G	Intron	*XRCC3*	rs3212038	0.1582	0.2901	0.3624	0.3361	0.2017	0.091
chr14	103699590	T	C	Intron	*XRCC3*	rs1799796	0.1579	0.2891	0.3584	0.3333	0.1999	0.5477
chr14	103706470	C	A	Intron	*XRCC3*	rs861531	0.2652	0.2664	0.08507	0.3754	0.2405	0.0136
chr14	103700738	C	T	Intron	*XRCC3*	rs861537	0.4698	0.5578	0.4226	0.7082	0.4332	0.2805
chr19	45408744	A	C	Intragenic	*ERCC1*	rs735482	0.2856	0.2733	0.4344	0.1405	0.2635	0.3047
chr19	45409148	G	A	Intragenic	*ERCC1*	rs2336219	0.2779	0.2728	0.4343	0.1404	0.2636	0.3065
chr19	45419065	G	T	Intron	*ERCC1*	rs3212961	0.2362	0.2584	0.4443	0.1286	0.2521	0.336
chr19	45409085	A	G	Intragenic	*ERCC1*	rs762562	0.2855	0.2730	0.4345	0.1403	0.2635	0.3047
chr19	45420395	A	G	Protein structural locus	*ERCC1*	rs11615	0.8862	0.7043	0.7344	0.3743	0.5293	0.8985
chr19	45368886	T	C	Intron	*ERCC2/XPD*	rs2298860	0.0008012	0.1631	0.008170	0.0002323	0.0005553	0.000
chr19	45351661	T	G	Nonsynonymous coding	*ERCC2/XPD*	rs13181	0.2251	0.1993	0.07552	0.3795	0.3715	0.1429
chr19	45364001	C	T	Nonsynonymous coding	*ERCC2/XPD*	rs1799793	0.1058	0.1956	0.04341	0.3545	0.3499	0.1191
chr19	45365033	T	G	Intron	*ERCC2/XPD*	rs1799785	0.0017	0.0934	0.1567	0.0008	0.0018	0.000
chr13	102858951	T	C	Intron	*ERCC5*	rs4150299	0.5741	0.3061	0.2633	0.5930	0.5020	0.2339
chr13	102872412	C	T	Intron	*ERCC5*	rs4150360	0.2319	0.3543	0.2088	0.1848	0.4366	0.1954
chr5	80654689	C	T	5′-UTR	*MSH3*	rs1105525	0.04785	0.1127	0.06772	0.1783	0.2405	0.000
chr5	80728800	T	G	Intron	*MSH3*	rs6151734	0.0005	0.01465	0.001345	0.0000	0.0008	0.0577
chr5	80672452	A	G	Intron	*MSH3*	rs1677653	0.2746	0.2230	0.02350	0.2631	0.2780	0.0136
chr5	80672439	T	A	Intron	*MSH3*	rs1650648	0.2740	0.2225	0.02310	0.2631	0.2785	0.0395
chr5	80654693	A	G	5′-UTR	*MSH3*	rs1105524	0.9187	0.6310	0.3927	0.6668	0.7514	0.4063
chr5	80854162	A	G	Nonsynonymous coding	*MSH3*	rs184967	0.8920	0.8667	0.9987	0.8429	0.8849	1
chr1	75912678	T	A	Intron	*MSH4*	rs28693610	0.1073	0.2917	0.5569	0.3903	0.5051	0.2059
chr1	75867478	T	G	Intron	*MSH4*	rs5745433	0.1600	0.2276	0.01961	0.2521	0.1856	0.0364
chr1	75803775	G	A	Nonsynonymous coding	*MSH4*	rs5745325	0.2978	0.2335	0.02654	0.2839	0.2133	0.0397
chr1	75867487	T	C	Intron	*MSH4*	rs3765682	0.0276	0.1524	0.2678	0.07137	0.05466	0.5167
chr1	226407946	C	G	5′UTR	*PARP1*	rs907187	0.05392	0.3036	0.4301	0.1631	0.1135	0.6855
chr1	226402008	C	T	Intron	*PARP1*	rs2666428	0.6124	0.4619	0.8060	0.3293	0.3771	0.6572
chr1	226392392	C	T	Intron	*PARP1*	rs2280712	0.05191	0.1076	0.3364	0.1682	0.1792	0.0136
chr1	226367601	A	G	Nonsynonymous coding	*PARP1*	rs1136410	0.05391	0.3020	0.4289	0.1593	0.1121	0.6588
chr1	226388595	G	A	Intron	*PARP1*	rs2293464	0.3651	0.1432	0.3374	0.1691	0.1771	0.0807
chr1	226388569	A	G	Intron	*PARP1*	rs2255403	0.1527	0.3101	0.4676	0.1596	0.1968	0.7032
chr1	226402132	T	C	Intron	*PARP1*	rs1805407	0.3885	0.1454	0.3373	0.1692	0.1798	0.0715
chr1	226385701	T	C	Intron	*PARP1*	rs1805408	0.3891	0.1453	0.3383	0.1691	0.1805	0.0794
chr1	226402257	G	A	Protein structural locus	*PARP1*	rs1805404	0.1248	0.4249	0.4472	0.1663	0.1030	0.000
chr1	226379307	C	G	Intron	*PARP1*	rs732284	0.3362	0.1388	0.3385	0.1689	0.1767	0.1016
chr14	20356854	C	T	Intron	*PARP2*	rs878157	0.09806	0.2069	0.2778	0.2703	0.3581	0.0143
chr14	20345513	A	G	Intron	*PARP2*	rs3093890	0.07983	0.1972	0.1907	0.2536	0.3426	0.0143
chr14	20357164	T	G	Intron	*PARP2*	rs200223594	0.1486	0.2147	0.2761	0.2765	0.3761	0.1084
chr14	20350953	T	A	Intron	*PARP2*	rs3093904	0.07983	0.1978	0.1918	0.2534	0.3440	0.0143
chr14	20357162	T	TTTCAC	Intron	*PARP2*	rs10625811	0.1490	0.2150	0.2767	0.2768	0.3762	0.113
chr14	20346854	C	T	Intron	*PARP2*	rs1713430	0.7030	0.4084	0.3690	0.3587	0.5194	0.4181

**Table 2:** Comparison between the allelic frequency of the study Native American populations (NAT) and five continental populations described in the gnomAD database for each variant investigated and their respective clinical impacts according to ClinVar.

Gene	SNPID	Variation Type	Impact	Molecular Function	NAT vs. AFR	NAT vs. AMR	NAT vs. EAS	NAT vs. EUR	NAT vs. SAS	ClinVar
*XPC*	rs1870134	SNV	Moderate	NER ^a^	0	0.0003	1	0	0	Xeroderma pigmentosum C
*XRCC3*	rs1799796	SNV	Modifier	HRR ^b^	0	0.0019	0.2189	0.0433	0	Breast cancer susceptibility
*TOP3A*	rs76300532	SNV	High	HRR ^b^	1	1	1	1	1	ND *
*ERCC1*	rs11615	SNV	High	NER ^a^	1	0.0146	0.0864	0	0	Efficacy/toxicity to carboplatin, cisplatin, oxaliplatin
*ERCC2/* *XPD*	rs13181	SNV	Moderate	NER ^a^	1	1	1	0.004	0.0065	Xeroderma pigmentosum D; Non-small-cell lung cancer and osteosarcoma
*ERCC2/* *XPD*	rs1799785	SNV	Modifier	NER ^a^	1	0.3452	0.0031	1	1	Xeroderma pigmentosum D
*ERCC5*	rs4150360	SNV	Modifier	NER ^a^	1	0.495	1	1	0.0034	Xeroderma pigmentosum G
*MSH3*	rs184967	SNP	Moderate	MMR ^c^	0.1433	0.0234	1	0.0033	0.056	Hereditary cancer-predisposing syndrome
*PARP1*	rs1805404	SNV	High	BER ^d^	0.0345	0	0	0.0012	0.1387	ND *

* ND = no data; ^a^ NER = nucleotide excision repair; ^b^ HRR = homologous recombination repair; ^c^ MMR = mismatch repair; ^d^ BER = base excision repair.

**Table 3:** New variants found in Native American population from Brazilian Amazon.

Chromosome (chr)	Position	Wild	Variant	Impact	Variation Type	Change Protein	Region Detailed	Gene	Molecular Function
chr10	68422310	T	A	Moderate	SNV	p.Glu871Val	Nonsynonymous coding	DNA2	BER
chr10	68422732	C	A	Moderate	SNV	p.Gln789His	Nonsynonymous coding	DNA2	BER
chr14	20346875	T	A	Moderate	SNV	p.Cys109Ser	Nonsynonymous coding	PARP2	BER
chr17	18271782	C	T	Modifier	SNV	c.* 3020G>A	3′-UTR	TOP3A	HRR
chr17	18314776	CA	C	High	INDEL	p.Met1fs	Frame shift	TOP3A	HRR
chr19	45351734	G	A	Modifier	SNV	c.2191-13C>T	Intron	ERCC2	NER
chr19	45364177	CA	C	Modifier	INDEL	c.815+57delT	Intron	ERCC2	NER
chr13	102865876	G	A	Moderate	SNV	p.Glu722Lys	Nonsynonymous coding	ERCC5	NER
chr5	80654908	G	GCAG…	Moderate	INDEL	p.Ala68_Pro69insProProAlaProProAlaProProAla	Codon insertion	MSH3	MMR

**Table 4:** Risk prediction of the new missense variants characterized in the study.

Chromosome (chr)	Position	Gene	Wild	Variant	Sequence	SIFT		PolyPhen	
chr10	68422310	DNA2	T	A	ENSG00000138346	0.03	Deleterious	0.506	Possibly damaging
chr10	68422310	DNA2	T	A	ENSG00000138346	0.08	Tolerated	0.877	Possibly damaging
chr10	68422310	DNA2	T	A	ENSG00000138346	0.05	Tolerated	0.909	Possibly damaging
chr10	68422310	DNA2	T	A	ENSG00000138346	0.35	Tolerated	0.694	Possibly damaging
chr13	102865876	ERCC5	G	A	ENSG00000134899	0.61	Tolerated	0.184	Benign
chr13	102865876	ERCC5	G	A	ENSG00000134899	0.6	Tolerated	0.024	Benign
chr13	102865876	ERCC5	G	A	ENSG00000134899	0.64	Tolerated	0.024	Benign
chr14	20346875	PARP2	T	A	ENSG00000129484	0.46	Tolerated	0	Benign
chr14	20346875	PARP2	T	A	ENSG00000129484	0.42	Tolerated	0.003	Benign
chr14	20346875	PARP2	T	A	ENSG00000129484	0.4	Tolerated	0	Benign
chr14	20346875	PARP2	T	A	ENSG00000129484	0.38	Tolerated	0.003	Benign

## Data Availability

The authors confirm that the data supporting the findings of this study are available within the article and its Supplementary Material. Raw data of the studied genes are available from the corresponding author, upon reasonable request.

## Supplementary Materials

The following supporting information can be downloaded at: https://www.mdpi.com/article/10.3390/genes13101869/s1, Supplementary Table S1 shows analysis on the 49 markers that are statistically significant, but not clinically significant according to the classification available in the ClinVar database (NCBI), nor classified as high-impact.
