# Supplementary material for: Molecular Epidemiology in Amerindians of the Brazilian Amazon Reveals New Genetic Variants in DNA Repair Genes
Source: Genes (Basel). 2022 Oct 15;13(10):1869. doi: 10.3390/genes13101869 (PMC9601515; doi:10.3390/genes13101869)
Supplement: Supplementary file 1 [file genes-13-01869-s001.zip › Table S1.pdf]

| Gene | SNPID | Variation Type | Impact | Molecular function | AFR | AMR | EAS | EUR | SAS | ClinVar |
|---|---|---|---|---|---|---|---|---|---|---|
| *DNA2* | rs10823209 | SNV | MODIFIER | BER | 0.5832 | 1 | **0.0017** | 1 | 1 | Not Reported in ClinVar |
| *DNA2* | rs34922453 | INDEL | MODIFIER | BER | 0 | **0.0021** | 1 | 0.0961 | 1 | Not Reported in ClinVar |
| *DNA2* | rs1801041 | SNV | MODIFIER | BER | **0.0119** | 0 | **0.0076** | 0 | 0 | Not Reported in ClinVar |
| *NEIL1* | rs11634109 | SNV | MODIFIER | BER | 1 | 1 | 1 | **0.0274** | 1 | Not Reported in ClinVar |
| *NEIL3* | rs7689099 | SNV | MODERATE | BER | 1 | **0.0234** | 1 | 0.0567 | 0.3393 | Not Reported in ClinVar |
| *NEIL3* | rs13112358 | SNV | MODERATE | BER | 0 | 0 | 1 | 0 | **0.0301** | Not Reported in ClinVar |
| *NEIL3* | rs13112390 | SNV | MODERATE | BER | 0.2706 | **0.0139** | 1 | **0.0015** | 1 | Not Reported in ClinVar |
| *TOP3A* | rs6502644 | SNV | MODIFIER | Homologous recombination | 0 | **0.003** | 1 | 0 | 0.175 | Not Reported in ClinVar |
| *TOP3A* | rs7207123 | SNV | MODIFIER | Homologous recombination | 0 | **0.001** | 1 | **0.0004** | 0.3747 | Not Reported in ClinVar |
| *TOP3A* | rs2294914 | SNV | MODIFIER | Homologous recombination | **0.0108** | 1 | 1 | **0.0192** | 1 | Not Reported in ClinVar |
| *TOP3A* | rs76300532 | SNV | HIGH | Homologous recombination | 1 | 1 | 1 | **1** | 1 | Not Reported in ClinVar |
| *TOP3A* | rs2294913 | SNV | MODIFIER | Homologous recombination | 0.384 | **0.001** | 0 | **0.0006** | 0.0727 | Not Reported in ClinVar |
| *TOP3A* | rs28671051 | SNV | MODERATE | Homologous recombination | **0.0007** | 1 | 1 | 1 | 1 | Not Reported in ClinVar |
| *TOP3A* | rs3817992 | SNV | MODIFIER | Homologous recombination | **0.0012** | 1 | 1 | 1 | **0.0207** | Not Reported in ClinVar |
| *XRCC3* | rs3212038 | SNV | MODIFIER | Homologous recombination | 1 | **0.0229** | **0.0001** | **0.0012** | 1 | Not Reported in ClinVar |
| *XRCC3* | rs861531 | SNV | MODIFIER | Homologous recombination | 0 | 0 | 1 | 0 | **0.0001** | Not Reported in ClinVar |

| | | | | | | | | | | |
|---|---|---|---|---|---|---|---|---|---|---|
| *XRCC3* | rs861537 | SNV | MODIFIER | Homologous recombination | 0.2201 | **0.001** | 1 | 0 | 1 | Not Reported in ClinVar |
| *ERCC1* | rs735482 | SNV | MODIFIER | NER | 1 | 1 | 1 | **0.0361** | 1 | Not Reported in ClinVar |
| *ERCC1* | rs2336219 | SNV | MODIFIER | NER | 1 | 1 | 1 | **0.0359** | 1 | Not Reported in ClinVar |
| *ERCC1* | rs3212961 | SNV | MODIFIER | NER | 1 | 1 | 1 | **8,00E-04** | 1 | Not Reported in ClinVar |
| *ERCC1* | rs762562 | SNV | MODIFIER | NER | 1 | 1 | 1 | **0.0356** | 1 | Not Reported in ClinVar |
| *ERCC2/XPD* | rs2298860 | SNV | MODIFIER | NER | 1 | **0.0019** | 1 | 1 | 1 | Not Reported in ClinVar |
| *ERCC2/XPD* | rs1799793 | SNV | MODERATE | NER | 1 | 1 | 0.5984 | **0.0055** | **0.0058** | Not Reported in ClinVar |
| *ERCC5* | rs4150299 | SNV | MODIFIER | NER | 0 | 1 | 1 | 0 | **0.0013** | Not Reported in ClinVar |
| *MSH3* | rs1105525 | SNV | MODIFIER | Mismatch repair | 1 | 0.0906 | 1 | **0.0007** | 0 | Not Reported in ClinVar |
| *MSH3* | rs6151734 | SNV | MODIFIER | Mismatch repair | 0 | 1 | **0.0003** | 0 | 0 | Not Reported in ClinVar |
| *MSH3* | rs1677653 | SNV | MODIFIER | Mismatch repair | 0 | **0.0003** | 1 | 0 | 0 | Not Reported in ClinVar |
| *MSH3* | rs1650648 | SNV | MODIFIER | Mismatch repair | **0.0006** | **0.0195** | 1 | **0.0011** | **0.0004** | Not Reported in ClinVar |
| *MSH3* | rs1105524 | SNV | MODIFIER | Mismatch repair | 0 | **0.0333** | 1 | **0.0023** | 0 | Not Reported in ClinVar |
| *MSH4* | rs28693610 | SNV | MODIFIER | Mismatch repair | 1 | 1 | 0 | 0.1706 | **0.0001** | Not Reported in ClinVar |
| *MSH4* | rs5745433 | SNV | MODIFIER | Mismatch repair | 0.2572 | **0.0027** | 1 | **0.0003** | **0.046** | Not Reported in ClinVar |
| *MSH4* | rs5745325 | SNV | MODERATE | Mismatch repair | **0.0001** | **0.0078** | 1 | **0.0002** | **0.0311** | Not Reported in ClinVar |
| *MSH4* | rs3765682 | SNV | MODIFIER | Mismatch repair | 0 | 0 | **0.0025** | 0 | 0 | Not Reported in ClinVar |

| | | | | | | | | | | |
|---|---|---|---|---|---|---|---|---|---|---|
| *PARP1* | rs907187 | SNV | MODIFIER | BER | 0 | 0 | **0.0035** | 0 | 0 | Not Reported in ClinVar |
| *PARP1* | rs2666428 | SNV | MODIFIER | BER | 1 | 0.2102 | 0.5624 | 0 | **0.0007** | Not Reported in ClinVar |
| *PARP1* | rs2280712 | SNV | MODIFIER | BER | 1 | 1 | 0 | **0.0149** | **0.0091** | Not Reported in ClinVar |
| *PARP1* | rs1136410 | SNV | MODERATE | BER | 0 | 0 | **0.0292** | 0 | 0 | Not Reported in ClinVar |
| *PARP1* | rs2293464 | SNV | MODIFIER | BER | 0 | 1 | **0.0002** | 1 | 1 | Not Reported in ClinVar |
| *PARP1* | rs2255403 | SNV | MODIFIER | BER | 0 | 0 | **0.0191** | 0 | 0 | Not Reported in ClinVar |
| *PARP1* | rs1805407 | SNV | MODIFIER | BER | 0 | 1 | **0.0002** | 1 | 1 | Not Reported in ClinVar |
| *PARP1* | rs1805408 | SNV | MODIFIER | BER | 0 | 1 | **0.0002** | 1 | 1 | Not Reported in ClinVar |
| *PARP1* | rs732284 | SNV | MODIFIER | BER | **0.0011** | 1 | **0.0007** | 1 | 1 | Not Reported in ClinVar |
| *PARP2* | rs878157 | SNV | MODIFIER | BER | 1 | **0.0013** | 0 | 0 | 0 | Not Reported in ClinVar |
| *PARP2* | rs3093890 | SNV | MODIFIER | BER | 1 | **0.0021** | **0.0033** | 0 | 0 | Not Reported in ClinVar |
| *PARP2* | rs200223594 | SNV | MODIFIER | BER | 1 | 1 | 0.1638 | 0.1672 | **0.0003** | Not Reported in ClinVar |
| *PARP2* | rs3093904 | SNV | MODIFIER | BER | 1 | **0.0021** | **0.0033** | 0 | 0 | Not Reported in ClinVar |
| *PARP2* | rs10625811 | INDEL | MODIFIER | BER | 1 | 1 | 0.163 | 0.167 | **0.0003** | Not Reported in ClinVar |
| *PARP2* | rs1713430 | SNV | MODIFIER | BER | **0.0003** | 1 | 1 | 1 | 1 | Not Reported in ClinVar |
